# Therapeutic drug monitoring in Parkinson’s disease

**DOI:** 10.1007/s00702-024-02828-5

**Published:** 2024-09-03

**Authors:** Thomas Müller, Manfred Gerlach, Gudrun Hefner, Christoph Hiemke, Wolfgang H. Jost, Peter Riederer

**Affiliations:** 1 Department of Neurology, St. Joseph Hospital Berlin-Weissensee, Gartenstr. 1, 13088 Berlin, Germany; 2 Department of Child and Adolescent Psychiatry, Psychosomatics and Psychotherapy, Center of Mental Health, University Hospital Würzburg, Würzburg, Germany; 3 Psychiatric Hospital, Vitos Clinic for Forensic Psychiatry, Kloster-Eberbach-Straße 4, 65346 Eltville, Germany; 4 Department of Psychiatry and Psychotherapy, University Medical Center of Mainz, Mainz, Germany; 5 Parkinson-Klinik Ortenau, Wolfach, Germany; 6 Center of Mental Health, Department of Psychiatry, Psychosomatics and Psychotherapy, University Hospital Würzburg, Margarete-Höppel Platz 1, 97080 Würzburg, Germany

**Keywords:** Therapeutic drug monitoring, TDM, Parkinson disease, Levodopa reference values, Dopaminergic receptor agonists reference values, Nutrition in Parkinson disease, Large neutral amino acids, Drug-drug interactions, Gastric emptying

## Abstract

A patient-tailored therapy of the heterogeneous, neuropsychiatric disorder of Parkinson’s disease (PD) aims to improve dopamine sensitive motor symptoms and associated non-motor features. A repeated, individual adaptation of dopamine substituting compounds is required throughout the disease course due to the progress of neurodegeneration. Therapeutic drug monitoring of dopamine substituting drugs may be an essential tool to optimize drug applications. We suggest plasma determination of levodopa as an initial step. The complex pharmacology of levodopa is influenced by its short elimination half-life and the gastric emptying velocity. Both considerably contribute to the observed variability of plasma concentrations of levodopa and its metabolite 3-O-methyldopa. These amino acids compete with other aromatic amino acids as well as branched chain amino acids on the limited transport capacity in the gastrointestinal tract and the blood brain barrier. However, not much is known about plasma concentrations of levodopa and other drugs/drug combinations in PD. Some examples may illustrate this lack of knowledge: Levodopa measurements may allow further insights in the phenomenon of inappropriate levodopa response. They may result from missing compliance, interactions e.g. with treatments for other mainly age-related disorders, like hypertension, diabetes, hyperlipidaemia, rheumatism or by patients themselves independently taken herbal medicines. Indeed, uncontrolled combination of compounds for accompanying disorders as given above with PD drugs might increase the risk of side effects. Determination of other drugs used to treat PD in plasma such as dopamine receptor agonists, amantadine and inhibitors of catechol-O-methyltransferase or monoamine oxidase B may refine and improve the value of calculations of levodopa equivalents. How COMT-Is change levodopa plasma concentrations? How other dopaminergic and non-dopaminergic drugs influence levodopa levels? Also, delivery of drugs as well as single and repeated dosing and continuous levodopa administrations with a possible accumulation of levodopa, pharmacokinetic behaviour of generic and branded compounds appear to have a marked influence on efficacy of drug treatment and side effect profile. Their increase over time may reflect progression of PD to a certain degree. Therapeutic drug monitoring in PD is considered to improve the therapeutic efficacy in the course of this devastating neurologic disorder and therefore is able to contribute to the patients’ precision medicine. State-of-the-art clinical studies are urgently needed to demonstrate the usefulness of TDM for optimizing the treatment of PD.

## Introduction

Therapeutic drug monitoring (TDM) is essential in epilepsy, but it has not yet been established for the clinical practice in patients with Parkinson’s disease (PD) (Hiemke et al. 2018). To enhance therapeutic efficacy and tolerability, determination of plasma levels of PD drugs recently gained more and more importance in the development of substances with an improved pharmacokinetic profile. Typical examples are extended release formulations of dopamine agonists, add on of amantadine and new intrajejunal or subcutaneous as well as inhalation formulations of levodopa particularly (Modi et al. 2019a; Rosebraugh et al. 2021; Marmol et al. 2021; LeWitt et al. 2022). The AGNP consensus guidelines for TDM already suggest therapeutic reference ranges of several antiparkinsonian drugs, only based on available pharmacokinetic properties of these neurologic drugs (Hiemke et al. 2018). In these guidelines, blood sample for several antiparkinsonian drugs is mostly suggested at t_max,_ the time of maximal drug concentrations (C_max_), as most of these drugs have a short elimination half-life, or if data at trough level conditions were not available. Nevertheless, TDM has not been established so far for antiparkinsonian drugs used in PD. This is surprising because PD is an aging-related devastating disorder, frequently combined with other diseases such as hypertonia, diabetes, hyperlipidaemia, rheumatism and others with their special drug treatment regimes.

## Nutritional aspects

Indeed, the pharmacokinetics of levodopa depend on several parameters, like health condition, nutritional aspects (interaction with protein-rich meals), gastrointestinal (GI) absorption and transfer via the blood–brain barrier). Levodopa competes with large neutral amino acids (phenylalanine, tyrosine, tryptophan, valine. leucine, isoleucine) for the mechanism of absorption and brain uptake (Cedarbaum 1987; Riederer 1980, Kessel and Aidy 2019).

Gastrointestinal barriers to levodopa transport and absorption include dysphagia, delayed gastric emptying, constipation, Helicobacter pylori infection, small intestinal bacterial overgrowth and gut dysbiosis (Leta et al. 2023). Levodopa is absorbed in the small intestine, where transit time is approximately three hours and plasma elimination half-life is short. Therefore, gastric emptying is a major determining factor for onset of symptom relief (Nyholm and Lennernäs 2008). Evidences on the gut microbiota-derived amino acid metabolism alterations associated with PD have been published by Wang et al. (2022).

Of note, specific gut bacteria might interfere with the bioavailability of levodopa (Kessel and Aidy 2019; Zhong et al. 2023; Beckers et al. 2022) as is the gastric acidity (Jenkins et al. 1973). Disorders of the gastrointestinal tract can adversely affect levodopa absorption and lead to Off-periods (Pfeiffer et al. 2020). GI disorders and GI resection may change the pharmacokinetics of levodopa (Nagayama et al. 2015). Drug-nutrient interactions between levodopa and enteral feedings are an important issue and may lead to decrease of clinical benefit of levodopa especially in critically ill patients (Cooper et al. 2008). Also, On–Off-phases in PD have been related to levodopa absorption and transport (Nutt et al. 1984). Notably, high protein meals or oral large neutral amino acids (LNAA) reverse the therapeutic effect of infused levodopa without reducing plasma levodopa concentration (Nutt et al. 1984; see also Molina et al. 1997; Pincus and Barry 1987). Application of subcutaneous and intestinal levodopa/carbidopa infusion systems is less complex and probably cheaper when combined with a COMT-I, i.e. opicapone with its one time daily intake scenario, from the beginning. It is well known that COMT-I reduce 3-OMD, which competes with levodopa transporter system, particularly at the blood brain barrier but also in the gastrointestinal tract in case of jejunal levodopa application. Therefore, nutritional interventions should be planned with regard to a nutritional balance designed to prevent weight loss or -gain, optimization of levodopa pharmacokinetics, avoidance of interactions with proteins and improvement in gastrointestinal dysfunction (Barichella et al. 2009). Indeed, protein restriction diets, low protein diet and levodopa administration long before any meals or even better after nutritional intake is considered to be a strategy to avoid decreased levodopa brain uptake (Barichella et al. 2009; Boelens Keun et al. 2021; Rusch et al. 2023; Eriksson et al. 1988; Murata 2006). The improvement in clinical response varied from 30% in patients with protein redistribution to 82% in those with low-protein diets (Virmani et al. 2016; Bracco et al. 1991). Indeed, small variations in plasma LNAAs in comparison with the large variations in plasma levodopa indicates that in this case LNAAs are of less importance to the fluctuating response to levodopa (Nutt et al. 1989). In addition, the metabolite of levodopa, 3-O-methyldopa, inhibits brain uptake of the parent compound (Nutt et al. 1987). Therefore, peripherally acting COMT-inhibitors are suggested to avoid this interaction.

## Drug-Drug interactions

For example, cardiovascular symptoms occur in PD and orthostatic hypotension is a frequent symptom (Jost 2020; Jost et al. 2020; Jost and Brück 2002). Of note, blood pressure in a 250 mg and 375 mg levodopa/benserazide group of older patients with PD in the early and middle stages, decreased significantly in the lying and standing positions (Su et al. 2023). However, arterial hypertension is also a symptom to be considered for drug therapy. Bitner et al. (2015) propose prescription of angiotensin-converting enzyme inhibitors, antagonists of AT1-receptor for angiotensin II, or antagonists of ß-adrenoceptors (ß-adrenolytics) as first-line agents of **antihypertensive treatment** in PD patients receiving levodopa /benserazide. Their analysis revealed a lack of clinically relevant drug-drug interactions (Bitner et al. 2015). In association with levodopa the dose of the potent antihypertensive drug guanethidine had to be reduced substantially (Hunter et al. 1970).

**Diuretics and beta-blockers** are still among the first-line medications for arterial hypertension, but both groups of substances should be used with caution in PD patients, among other things because of orthostatic hypotension (Jost and Brück 2002; Lee et al. 2014).

In the MPTP-treated two monkeys an intraperitoneal injection of the ß-adrenergic agonist isoproterenol increased the blood-to-brain transport of levodopa (Alexander et al. 1994).

**Anticholinergic drugs** markedly delay gastric emptying and thus may reduce the therapeutic benefit from levodopa (Bianchine and Sunyapridakul 1973).

Potential drug-drug interactions in hospitalized patients with PD exhibiting multiple comorbidities, hospitalized in a neurodegenerative ward and/or taking antidepressants or antipsychotic drugs, with levodopa can be expected (Aleksic et al.^2021^). Caution is recommended when antidepressants are given in combination with monoamine oxidase inhibitors (Nieuwstraten et al. 2006; Edwards 1982; Chen et al.^2023^). However, this primarily affects MAO-A inhibitors and only to a very limited extent the MAO-B inhibitors used in PD.

**Monoamine oxidase inhibitors** (MAO-Is) belong to the earliest drugs tried in PD with and without levodopa (Bernheimer et al. 1962). Due to their side effect/adverse reaction profile unspecific MAO-Is have limited use in the treatment of depression in PD (Teychenne et al. 1975), while selective, reversible MAO-A inhibitors are recommended (Riederer and Laux 2011), The selective, irreversible MAO-B inhibitors selegiline and rasagiline are used to treat akinesia and motor fluctuations as is with the selective, reversible MAO-B inhibitor safinamide. Selegiline has been marketed as tablet and melting tablet for PD and as transdermal selegiline for depression, while rasagiline and safinamide are recommended for the treatment of PD (Riederer and Laux 2011; Flockhart 2012; Dezsi and Vecsei 2017; Tabi et al. 2020; Asano 2023; Robakis and Fahn 2015; Krishna et al. 2014). Concurrent administration of selegiline and **selective serotonin uptake inhibitors** should be avoided due to the possible development of the serotonin syndrome (Ritter and Alexander 1997; Frenklach 2016). Hypertensive crises and the serotonin syndrome can occur in rare cases due to interactions between MAO-Is and food containing tyramine as well as interactions with serotoninergic and sympathomimetic agents (Flockhart 2012; Livingstone and Livingstone 1996). No dietary restrictions, however, are imposed and no hypertensive crises result when MAO-B inhibitors are used in combination with levodopa (Flockhart 2012; Riederer and Laux 2011).

**Pyridoxine** (Vitamin B6) has been shown to decrease the effects of levodopa. However, this effect is not seen when levodopa is prescribed in combination with a decarboxylase inhibitor (Mars 1974; Sulli and Ezzo 2007).

Diabetes has been shown also to be frequently associated with PD (Sandyk 1993). Mainly type 2 diabetes mellitus is a risk factor for the onset and progression of both, Alzheimer’s disease and PD (Cardoso and Moreira 2020). Therefore, **antidiabetic drugs** are administered and might interfere with levodopa treatment even in pancreatic cells. Of interest, metformin in the 6-hydroxydopamine-lesioned mouse model of PD normalized the increased glycogen synthase kinase-3ß activity induced by chronic treatment with levodopa. Thus, metformin may have therapeutic potential for the suppression or management of levodopa-induced motor complications in patients with PD (Ryu et al. 2018), but see Ping et al (2020). Intranasal insulin, metformin and thiazolidinediones are used in PD (Cardoso and Moreira 2020).

**Cholesterol-lowering drugs** are used in PD to antagonize enhanced cholesterol or lipid-related disorders in order to improve cognitive abilities (Roy and Pahan 2011). In their experimental study in a chronic MPTP-model of PD Roy and Pahan (2011) demonstrated that simvastatin, which crosses the BBB and negatively correlates with the incidence of PD, shows efficacy in this animal model of PD.

The disease-modifying **antirheumatic drugs** (DMARDs) chloroquine/hydroxychloroquine have been shown to be associated with a decreased risk of PD (Paakinaho et al.^2022^), whereas centrally acting calcium antagonists, i.e. amlodipine, are at least under suspicion to influence progression respectively the risk for PD onset (Lee et al. 2014; Ritz et al. 2010; Sempere et al. 1995).

Patients taking levodopa suggest that digoxin, diuretics, phenindione, cyclizin, dichloralphenazone, barbiturates, general anaesthetics, chlorpropamide, insulin, dexamphetamines, ampicillin, sulphadimidine, antacids, prednisolone, thyroxine and paracetamol can safely be given in association with levodopa (Hunter et al. 1970).

Uncontrolled combination of all these drugs with antiparkinsonian medications might interfere with levodopa, plasma concentrations and may increase the risk for side effects as there exists an inter individual pharmacokinetic variability of the drugs in patients (Preskorn et al. 1993; Preskorn 2008). TDM is able to optimize drug treatment on a personal basis, reduces the risk for drug-drug interactions substantially, controls dose regimes, proves compliance, contributes to clarify non-response at therapeutic doses, improves drug adherence and suboptimal tolerability. In the case of fos-levodopa/fos-carbidopa administration, change of dosing of alkaline phosphatase enzyme activity influencing drugs, such as antidiabetics or anticonvulsants, may influence the dephosphorylation capacity of fos-levodopa/fos-carbidopa to L-dopa/carbidopa.

Therefore, TDM in PD is helpful to optimize “precision medicine” in this disorder. Indeed TDM has been shown since decades to improve the therapeutic outcome on an individual basis in neuropsychiatric patients (Adli et al. 2005; Trivedi et al. 2007; Ceskova 2014; de LJ 2014; Shin et al. 2016). Therefore, it is our aim to further stimulate the idea to use TDM in optimizing treatment strategies particular in PD but also in neurology in general. Notably, methodology for the calculation of reference values can be followed in the detailed description by Hiemke et al. (2018). An update can be expected in 2024/2025.

## Levodopa

The current scientific interests particularly focus on monitoring levodopa in plasma. Levodopa is the most efficacious PD drug and well tolerated. Its short half-life lasts between 30 to 90 min. This fast metabolism rate gives rise to fluctuations of levodopa plasma concentrations over the day. The peripheral rise and fall of levodopa after oral administration in plasma is translated into pulsatile dopamine brain levels after the levodopa transport via the blood brain barrier and its presynaptic dopa decarboxylase mediated conversion to dopamine. Such levodopa-induced fluctuations of dopamine in the nigrostriatal system counteract the physiological principle of continuous dopaminergic stimulation. Studies of the normal function of basal ganglia interaction have demonstrated that nigral dopaminergic neurons normally fire continuously and that striatal dopamine concentrations are relatively constant (Stocchi 2009). In MPTP treated monkeys, pulsatile stimulation of striatal dopamine receptors with short-lasting agents is associated with the induction of molecular and physiologic changes in basal ganglia neurons and the development of motor complications (Stocchi 2009; Chase 1998). Therefore, continuous delivery of levodopa might be associated with a reduced risk of motor complications (Olanow et al. 2020; Pirtosek et al. 2023; van Warmelen et al. 2018; Nutt et al. 2000; Nyholm 2007; Metman et al. 2001). Nevertheless, the understanding of the mechanisms behind the complications of long-term levodopa treatment (especially in advanced PD) is still not complete (Timpka et al. 2016; Antonini 2007). The way in which drugs are delivered in PD appears to have a marked influence on both efficacy and side effect profile. Therefore, the concept of “continuous drug delivery” has been arisen (Gershanik and Jenner 2012).

This paradigm of dopamine substitution is regarded as an essential component for adequate motor behaviour in PD patients with loss of motor function and motor complications. The increase in loss of compensating capacity is due to the progression of PD. This balance decline eases onset of fluctuations of movement and associated non motor symptoms, both of which are mainly related to the oral application of levodopa/ peripheral dopa decarboxylase inhibitor (DDI) formulations. Thus, they are looked upon as mainly responsible for the pulsatility of levodopa, or brain dopamine, under repeated oral dosing. Accordingly, the so-called fluctuation index (FI) of levodopa is looked upon especially for scientific reasons (Ferreira et al. 2013; Müller 2013) as a measure of the magnitude of rise and fall of levodopa in plasma relative to the average concentration. The lower the FI, the more the maximum levodopa concentration (C_max_) is associated to the plasma levodopa trough (C_min_). Therefore, in the long term, a low FI may minimize onset of potential C_max_ related adverse effects, such as dyskinesia. These involuntary movements are the consequence of an over stimulation of the progressed degenerated nigrostriatal dopaminergic system with levodopa. Accordingly, clinical researchers focused on the pharmacokinetic behaviour of orally applied levodopa preparations following single and repeated dosing. Their results underlined the importance of measuring levodopa plasma concentrations to control the fluctuations. A reference value Cmax of 900 -2000 ng/ml has been reported (Hiemke et al.^2018^) suggesting rather a rough value of expectation. Table 1 only reports a selection of pharmacokinetic studies on levodopa/DDI formulations with PD patients (except: Rosebraugh et al. 2022). Body weight and inhibition of levodopa metabolizing enzymes, such as catechol-O-methyltransferase (COMT), may contribute to the observed data variability, which also results from an individually differing gastrointestinal absorption and genetic individuality (Fermaglich and O'Doherty 1972; Nyholm & Lennernas 2008; Muller 2013; Lipp et al. 2016; Leta et al. 2023). Data from healthy volunteers should not be considered because chronic dopamine substitution interferes with gastrointestinal motility (Fermaglich and O'Doherty 1972). It causes a delay of gastric emptying. Chronic dopamine substitution with gastrointestinal dopamine receptor stimulation delays absorption of an oral levodopa formulation in the duodenal tract. Therefore, pharmacokinetic data of healthy volunteers may considerably differ from long term levodopa treated PD patients. Additionally, pharmacokinetic data (not reference values) reported in Table 1, also mirror the fact that the performance of investigations, or the reporting of results in the literature on levodopa in plasma are not standardized. Therefore, these data can be used to further improve the Cmax reference values in an updated version of the Hiemke et al. (2018) publication, as suggested for 2024/2025. Some trials reported median-, others mean values. Area-under-the-curve (AUC) outcomes were calculated over differing, sometimes over intervals which were not even mentioned. In case of repeated drug intake, varying intake times were chosen. A further problem is response to levodopa, as no real objective and reliable assessment of the impaired motor behaviour is possible in treated PD patients, even after an overnight withdrawal of dopamine substituting compounds. Indeed, concomitantly applied dopamine substitution, i.e. with dopamine agonists that possess a half-life of up to 24 h, may considerably bias the clinical assessment of motor symptoms. The situation is similar when PD drugs are applied in a continuous fashion by a patch (rotigotine) or as an infusion (levodopa intestinal gel, ND0612 or foslevodopa). Thus, the situation differs from other neurological, or psychiatric conditions in clinical practice, i.e., in epilepsy with the possibility of objective electroencephalography recordings to determine the likelihood of seizures. Therefore, TDM may indeed be helpful to optimize drug treatment in PD.

**Table 1:** Pharmacokinetic data but not reference values of various available levodopa containing formulations

		C_max_		T_max_		AUC				Reference
		Mean (ng/ml)	SD	Mean (min)	SD	Mean (h * ng/ml)	SD	Number of measurements in individual patients	Intake	
**Immediate release formulations**										
Sinemet® immediate release	100 mg Levodopa/ 25 mg Carbidopa	167	86	38	63.6	13,192 hour: 3	279	94	1	(Contin et al. 2021) computed from the original data set
Sirio® immediate release	100 mg Levodopa/ 25 mg Carbidopa	177	18	30	21.2	12,266 hour: 3	508	7	1	(Contin et al. 2021) computed from the original data
Madopar ® immediate release	100 mg Levodopa/25 mg Benserazide	211	17,2	60	42.4	14,802 hour: 3	686	242	1	(Contin et al. 2021) computed from the original data set
Levodopa-benserazide Teva 200–50® immediate release	100 mg Levodopa/25 mg Benserazide	198	60	45	22.6	13,708 hour: 3	317	56	1	(Contin et al. 2021) computed from the original data set
Sinemet® immediate release	100 mg Levodopa/25 mg Carbidopa	121	15 (SEM)	Not reported		45,220 hour: 8	676 (SEM)	18	3	(Olanow et al. 2019)
Sinemet® immediate release	100 mg Levodopa/25 mg Carbidopa	236	110	0.9 h	0.5 h	Not reported	Not reported	27	1	(Hauser et al. 2011)
Sinemet® immediate release	100 mg Levodopa/25 mg Carbidopa	141	65			Not reported	Not reported		Every 3 h over 8 h interval	(LeWitt et al. 2022)
Sinemet® immediate release	100 mg Levodopa/25 mg Carbidopa	236	117	Not reported	Not reported	Not reported	Not reported	27	1	(Modi et al. 2019b)
Sinemet® immediate release	100 mg Levodopa/25 mg Carbidopa	249	Not reported	Not reported	Not reported	Not reported	Not reported	26	1	(Modi et al. 2019b)
Sinemet® immediate release	100 mg Levodopa/25 mg Carbidopa	187	70	0.5	0.5–1.5	31,730	1090	11	1	(Mittur et al. 2017)
Sinemet® immediate release	200 mg Levodopa/50 mg Carbidopa	233	77	1.3	0.5–2	59,780	1830	10	1	(Mittur et al. 2017)
Sinemet® immediate release	250 mg Levodopa/75 mg Carbidopa	394	157	0.5	0.5–1	76,900	1090	4	1	(Mittur et al. 2017)
**Extended release**										
Sinemet® extended release	200 mg Levodopa/50 mg Carbidopa	145	81	2	1–4	50,000	3070	4	1	(Mittur et al. 2017)
**Soluble levodopa formulation**										
Madopar dispersible®	100 mg Levodopa/25 mg Benserazide	201	86	29	11	14,727 hour: 3	451	14	1	(Contin et al. 2021) computed from the original data set
Sinemet® as solution dissolved 50 ml neutral pH water without buffer and administered as a sip	100 mg Levodopa/25 mg Carbidopa	115	164 (SEM)	Not reported		43,870 hour: 8	609 (SEM)			(Olanow et al. 2019)
**New galenic extended release formulation**										
IPX066		300	130	2 h	1.1 h	Not reported	Not reported	27	1	(Hauser et al. 2011)
IPX066	100 mg Levodopa/25 mg Carbidopa	284	Not reported	Not reported	Not reported	Not reported	Not reported	26	1	(Modi et al. 2019a)
Rytary ® (IPX066)	435 mg Levodopa/108.75 carbidopa	169	97	1.5 h (median)	1.5–8 (range)	Not reported	Not reported	12	1	(Mittur et al. 2017)
Rytary ® (IPX066)	490 mg Levodopa/122.5 mg carbidopa	242	76	2.5 h	0.5–4	11,291	3945	16	1	(Mittur et al. 2017)
Rytary ® (IPX066)	585 mg Levodopa/146.25 carbidopa	294	136	1.5	0.5–2	15,210	8905	7	1	(Mittur et al. 2017)
Rytary ® (IPX066)	735 mg Levodopa/183.75 mg carbidopa	309	70	1.5	0.5–3	13,221	3756	14	1	(Mittur et al. 2017)
Rytary ® (IPX066)	780 mg Levodopa/183.75 mg carbidopa	370	122	2	0.5–3	18,424	4093	3	1	(Mittur et al. 2017)
Rytary ® (IPX066)	980 mg Levodopa/183.75 mg carbidopa	497	104	2	0.5–4	20,479	3257	6	1	(Mittur et al. 2017)
**Immediate release and new galenic extended release formulation**										
IPX203	100 mg Levodopa/25 mg Carbidopa	277	126	Not reported	Not reported	Not reported	Not reported	27	1	(Modi et al. 2019b)
IPX203	100 mg Levodopa/25 mg Carbidopa	316	Not reported	Not reported	Not reported	Not reported	Not reported	26	1	(Modi et al. 2019a)
**Subcutaneous infusion**										
ND0612004	ND0612 60 mg levodopa/7.5 mg carbidopa/ml rate: 0.64 ml/h	136	27	Not reported	Not reported	6466	1404	7	Infusion	(LeWitt et al. 2022)
**Subcutaneous phosphorylated levodopa and phosphorylated carbidopa infusion**										
foslevodopa	Foslevodopa	658	Not reported	Not reported	Not reported	Not reported	Not reported	Healthy volunteers		(Rosebraugh et al. 2022)
**Intrajejunal levodopa/carbidopa infusion**										
	LCIG	187	Not reported	Not reported	Not reported	Not reported	Not reported	Healthy volunteers		(Rosebraugh et al. 2022)
**Not described in detail**										
	100 mg L-dopa with 25 mg benserazide or 25 mg carbidopa	227 mg/ml	16 SEM)	46.4	26.8	Not reported	Not reported	14	1	(Adamiak-Giera et al. 2021)
	100 mg L-dopa with 25 mg benserazide or 25 mg carbidopa and ropinirol	179	95 (SEM)	35.5	34.6	Not reported	Not reported	13	1	(Adamiak-Giera et al. 2021)

Concentrations are given as ng/ml in case of mean or median of maximum concentration (C_max_) measured at T_max_, respectively as h * ng/ml in case of Area Under the Curve (AUC) values; SD, standard deviation, SEM, standard error of means; interval to maximum concentration (T_max_) is given in minutes, computed by descriptive analysis from the original data set of Contin et al. 2021

## Reliable reference ranges are currently under investigation

For levodopa the AGNP-TDM consensus guideline defines a reference range of 0.9–2.0 µg/mL in blood sample 1 h after 250 mg levodopa combined with 25 mg carbidopa (Hiemke et al. 2018). For levodopa, a moderate correlation between drug concentrations in blood and short-term clinical response is considered (Nutt & Fellman 1984). Overall, however, the literature is not consistent in terms of reference ranges with upper and lower threshold values as an essential precondition for an adequate motor response (Contin et al. 1999; Stocchi et al. 2005; Contin & Martinelli 2010). One may hypothesize that continuous subcutaneous delivery of levodopa/carbidopa (ND0612 or foslevodopa) will probably need lower levels in PD patients. Infusion systems circumvent the gastrointestinal tract. The principle of continuous levodopa administration with a possible accumulation, or a cautious titration to a steady state of plasma levodopa, may represent an essential component for the efficacy of the drug after its active transport over the blood brain barrier. Therefore, reliable reference values are currently under investigation. Accordingly, determinations of plasma levodopa in a TDM setting may gain more importance in the near future. In this respect a more standardised calculation of levodopa plasma levels may allow clinicians to differentiate better between branded and generic levodopa formulations. In the past years the impact of health care authorities, -politicians and payers went up in terms of drug cost reduction. Various approaches of not transparent price regulation scenarios of country specific healthcare system increasingly influence drug selection and accordingly limit therapeutic options. Generally this system supports prescription of cheaper, generic available drugs, however these compounds considerably vary in their pharmacokinetic behaviour. In Germany, even the pharmacist may switch between generic and branded compounds without information of the prescribing neurologists. Therefore, the efficacy of a personalised PD drug treatment cocktail may change, particularly, when compounds with a short half-life are applied. Therefore, effects of oral levodopa formulations are particularly altered by differences between original and the various generic available oral levodopa formulations. This scenario is due to country specific, sometimes even regional different, not transparent drug price reduction- and discount procedures. It is supplemented by direct or hidden modes of budget limitations for prescribing neurologists, i.e. in Germany. Therefore, TDM for the various levodopa formulations in a defined, strictly standardised fashion will particularly gain importance in the future to counteract this currently ongoing development, which may provide harm to patients even in terms of drug safety.

## The impact of COMT

Enzymatic blocking of levodopa metabolism with COMT-inhibitors (COMT-I) additionally influences plasma bioavailability of levodopa. Table 2 shows that COMT-I increases the median levels of levodopa. Median concentrations are here reported, as they are not so sensitive to outliers compared to means. Data were taken from previously published pharmacokinetic trials (Müller et al. 2006; Muhlack et al. 2014; Müller et al. 2022). These investigations have in common that PD patients received no other PD drug overnight, only 100 mg levodopa/25 mg carbidopa with and without COMT-inhibitor in the currently approved and thus applied dosing (200 mg entacapone with each levodopa dose, or tolcapone 100 mg t.i.d. or 50 mg opicapone o.i.d) and showed a positive motor response to the levodopa administration during the investigation interval. Only the outcomes of the determinations at baseline, 60, 120 and 180 min after drug intake were taken for this analysis, as these moments were the same in all these trials. Co-medication with 50 mg opicapone with its more consistent COMT-blocking enhanced the median AUC levels of levodopa by 370% compared to 55 and 53% with addition of 200 mg entacapone and 100 mg tolcapone, respectively (Table 2). Effects on C_max_ showed a tendency for an increase with 200 mg entacapone and 100 mg tolcapone only but a substantial one for 50 mg opicapone. The SEM values reflect the high variability of levodopa in plasma bioavailability across PD patients (Müller, 2010a; b). As aforementioned, branded and generic oral levodopa formulations considerably differ in terms of pharmacokinetic behaviour. Authorities define bio equivalence with differences within the range of 80% and 125% of the original formulation. Therefore, the dispersion width of the various available generic formulations varies up to 45% (Müller, 2022). If they are introduced by the pharmacist only, as in Germany, the efficacy of a patient tailored PD drug regimen can markedly decline. However, clinicians point out that effects of oral levodopa applications are considerably influenced by even minimal differences between original and the various generic oral levodopa formulations (see Table 1) (Müller, 2022). This can easily be shown by measuring plasma levels of levodopa. Additionally, genetic subtypes of plasma transporter proteins, such as orosomucoid, may contribute to the variability of levodopa plasma concentrations (Weidinger et al. 1987). Therapeutic reference ranges of entacapone (0.4–1.0 µg/ml 1 h after intake) and tolcapone (3–6 µg/ml 2 h after intake) are proposed in the AGNP guidelines, based solely on pharmacokinetic properties (Hiemke et al. 2018). However, as COMT-Is are not administered without levodopa, TDM of COMT-Is is not recommended.

**Table 2:** Pharmacokinetic data but not reference values of the impact of COMT inhibitors on the pharmacokinetics of levodopa

	AUC	C_max_	T_max_
100 mg levodopa/25 mg carbidopa (Nacom 100 ®)			
Median	22,293	215	60
SD	3212	30	6.4
100 mg levodopa/25 mg carbidopa/200 mg entacapone (Stalevo 100®)			
Median	34,544	283	60
SEM	5818	44	5.6
100 mg levodopa/25 mg carbidopa plus 100 mg tolcapone (Nacom 100® plus Tasmar®)			
median	34,058	290	60
SEM	5479	48	9.9
100 mg levodopa/25 mg carbidopa plus 50 mg opicapone (Nacom 100® plus Ongentys ®)			
median	104,955	1053	30
SEM	18,355	158	3.9

Concentrations are given as ng/ml in case of mean or median of maximum concentration (C_max_) measured at T_max_, respectively as h * ng/ml in case of Area Under the Curve (AUC) values; SEM, standard error of means; interval to maximum concentration (T_max_) is given in minutes. Cave: we took the reported values from the original publications. There is no consistency in terms of statistical values. We emphasize, that it is not ideal to report both mean + -SD resp. SEM values and Median/Interquartile range in the same table. However, we had no access to the original data set in order to re-calculate statistics

## Dopamine agonist plasma levels in PD patients

It is mandatory for approval of a dopamine agonist (DA) to provide pharmacokinetic data, including bioavailability and elimination half-life. Pharmacokinetic trials, however, are usually performed in healthy volunteers. Comparisons between approved and new delivery formulations are also common even in PD patients, (for an example see Hattori et al. 2012). They aim to demonstrate non inferiority in terms of bioavailability and absence of a significant impact on levodopa pharmacokinetics (Deleu et al. 2002; Tompson & Vearer 2007). One of the best and most investigated compound is rotigotine because of its transdermal application route (Montastruc et al. 1991; Nicolle et al. 1993; Persiani et al. 1994; van et al., 1995; Kaye & Nicholls 2000; Hubble & Novak 2001; Del & Bonuccelli 2003; Elshoff et al. 2012; Cawello et al. 2014; Liu et al. 2018). A second rotigotine patch is now on the market, which differs in both the patch size and the rotigotine load (Jost et al. 2024). The data situation is currently not sufficient. Generally the clinical pharmacokinetics of ergoline and non-ergoline DA are poorly characterized in PD patients (Friis et al. 1979). In one trial, blood sample for measurement of DA plasma concentrations were drawn in the morning at a median 18 h-distance from the last DA intake with pramipexole, ropinirole or transdermal rotigotine (the latter does not show a first-pass effect). The high inter subject variability in plasma concentrations under the same dosage may implicate that monitoring DA plasma levels could also be helpful in the individualization of treatment (Contin et al. 2019). However, both data on pharmacokinetic effects of the DA on levodopa or valid therapeutic ranges under steady state conditions, are not available for the clinical practice in PD patients, i.e. to control expected absorption problems. For piribedil, a reference value of 10–30 ng/ml was proposed (Ziegler et al. 1994; Montastruc et al. 1999). A reference value between 4 and 10 ng/ml was mentioned for apomorphine (Agbo et al. 2021; Kim et al. 2022). The AGNP guidelines published the following reference ranges: for bromocriptine (low dose (2.5 mg): 0.1–0.3 ng/ml; max. dose (25 mg): 1.0–4.0 ng/ml; trough level), cabergoline (58–144 pg/ml 0.5–4 h after drug intake for 4 weeks), pramipexole (0.4–7.2 ng/ml; trough-level), ropinirole (0.4–6.0 ng/ml; trough-level) and rotigotine (0.1–0.7 ng/ml; trough-level),.

However, dopamine receptor agonists at optimum doses to improve motor behaviour of patients with PD have lost importance. This is due to their increasing neuropsychiatric side effect profile as PD progresses. As a consequence, these drugs are used at low doses in the early phase of PD only, but are used most frequently as add-on medication to levodopa treatment. In this context, TDM of dopaminergic receptor agonists is not helpful.

## Anticholinergic/-muscarinergic PD drugs

Amelioration of tremor in PD with anticholinergic compounds is nowadays rarely performed. To our best knowledge clinical pharmacokinetics or therapeutic levels of anticholinergics in PD patients are scarcely available. Solely the AGNP consensus guidelines published a therapeutic reference range for biperiden (1–6.5 ng/ml 0.5-2 h after intake of 4 mg) and bornaprin (0.7–7.2 ng/ml 1–2 h after intake of 4 mg) (Hiemke et al. 2018). No relevant impact of administration of anticholinergics on levodopa pharmacokinetics has been described similar to dopamine agonists, i.e. ropinirole (Contin et al. 1991; Deleu et al. 2002; Adamiak-Giera et al. 2021).

## Drug monitoring of amantadine

Three mechanisms of action might explain the efficacy of amantadine in PD. Preclinical data demonstrate differential modulation of the dopamine system. Inhibition of dopamine reuptake and stimulation of central dopa-decarboxylase activity may be clinically relevant, as they support dopaminergic neurotransmission. Accordingly, presynaptic and postsynaptic actions of amantadine at dopaminergic terminals and release of intraneuronal dopamine from extravesicular stores have been reported. Amantadine also inhibits the N-methyl-D-aspartate (NMDA) subtype of glutamate receptors particularly during high dosing. A mild anticholinergic effect is also well known (Fisher et al. 1998; Deleu et al. 2002; Danysz et al. 2021). In view of these complex pharmacologic and thus also pharmacodynamic effects it is secondary to establish TDM for amantadine in clinical practice. A therapeutic reference range of amantadine (300–600 ng/ml, trough level) has been proposed (Hiemke et al. 2018), but there is no data for both, amantadine HCL and amantadine SO_4_.

## Monoamine oxidase B inhibitors

Levodopa is transformed by dopa-decarboxylase to dopamine within dopamine synthesizing neurons mostly. Central dopamine turnover is predominantly catalysed by the enzymes COMT and MAO in neuronal and glial cells. MAO is located in the outer mitochondrial membrane. Two enzyme forms of MAO exist. They are classified as MAO-A and MAO-B and differ in terms of preferred substrates. MAO-A mostly degrades serotonin and norepinephrine in the brain, placenta, gut and liver. MAO-B metabolizes preferentially phenylethylamine, methylhistamine and tryptamine in glial brain cells neighbouring dopaminergic synapses, liver and platelets. Both MAO subtypes are also involved in tyramine- and dopamine metabolism (Tabi et al. 2020). MAO-inhibitors generally reduce the turnover of biogenic amines. Inhibition of one MAO isoform activity is only enabled within a rather low concentration range of the applied drug. This specific inhibition pattern changes with higher drug dosing. Then a total blockade of both MAO isoforms occurs. Thus MAO-B enzyme activity contributes to regulation of storage, release and occurrence of free concentrations of biogenic amines in the synaptic cleft. There are three compounds available. Selegiline is quickly absorbed in the gastrointestinal tract. Main derivatives of selegiline are desmethylselegiline, methamphetamine and L-amphetamine. Selegiline is applied once or twice daily, in 5 mg tablets up to 10 mg, mostly 7.5 mg are administered (Birkmayer et al. 1975). Rasagiline is also well absorbed (Finberg & Youdim 2002). one hour following administration of an oral rasagiline formulation, inhibition of MAO-B activity was found in platelets. This effect lasted for at least 48 h and then returned to baseline after two weeks. Rasagiline is degraded to aminoindane. Rasagiline is orally applied once daily in a dosage of 1 mg. Safinamide is a reversible MAO-B inhibitor and selegiline and rasagiline are irreversible inhibitors. Safinamide is well and quickly absorbed in the gastrointestinal tract. The maximum plasma concentration was observed within 2–4 h after oral intake. Extensive metabolism of safinamide via amide hydrolytic oxidation generates safinamide acid as main derivative. Further less important metabolites are formed, such as O-debenzylated safinamide. Safinamide is administered in a dosage of 50 or 100 mg once daily (Müller and Foley 2017). TDM with MAO-B inhibitors will only make sense in terms of the determination of MAO-B enzyme inhibition for the clinical practice, as the enzyme inhibition considerably varies as shown in the periphery (Müller et al., 2017; Riederer and Müller 2018). Furthermore, for irreversible MAO-B inhibitors, drug concentrations in blood do not correlate with clinical effects due to irreversible blockade of an enzyme. Therefore, TDM is not recommended for dose titration, but may be potentially useful for special indications or problem solving (Hiemke et al. 2018).

## Outlook

Currently, therapeutic drug monitoring is not a well-developed research field for the treatment of PD patients in contrast to other disease entities, such as epilepsy. Though TDM has the potential to improve efficacy and tolerability of PD drugs, it is so far not used in clinical practice. For example, antiparkinsonian drugs exhibit concentration dependent properties, and TDM may detect non-adherence or pharmacokinetic abnormalities due to genotype or pharmacokinetic drug-drug interactions (Hiemke et al. 2018). To our best knowledge, COMT gene mutations have no impact on levodopa plasma levels (Muller 2010a, b). As aforementioned, the main investigations on plasma bioavailability of levodopa are available in PD and summarized in Table 1. This table also shows that AUC- and C_max_ values considerably differ in pharmacokinetic trials, even when the same compound, i.e. immediate release Sinemet® (100 mg levodopa/25 mg carbidopa) were administered. Therefore a standardized procedure for determination of plasma levodopa is essential. The cited references also have in common that they do not even consider the existing rough clinical differentiation of PD in subtypes with predominant tremor or akinesia and rigidity or equivalent expression of akinesia, rigidity and tremor. Treatment of these subtypes may differ in terms of tremor amelioration, i.e. with antimuscarinic compounds. They may influence gastrointestinal motility and thus finally delay levodopa absorption after its transport from the stomach to duodenal structures, where levodopa absorption predominantly takes place. Moreover, PD related syndromes, such as progressive supra nuclear palsy or multi system atrophy, do in fact exist. Their diagnosis-complementing features also include a weakened or even missing levodopa response in addition to a vast and broad literature on neuropathological and morphological imaging findings. However to date, reliable investigations on the gastrointestinal absorption and plasma bioavailability of levodopa, or its transport via the blood brain barrier with levodopa- and dopamine determinations in cerebrospinal fluid, are rare (Veronneau-Veilleux et al. 2020, 2021; Ursino et al. 2020). Simultaneous levodopa assessment in plasma and cerebrospinal fluid may also serve as a future capacity marker of blood brain barrier transporters to shuttle biogenic amines into the brain and for the central regulation of the central turnover of levodopa to dopamine via central dopa-decarboxylase in addition to these more hypothetical model calculations (Veronneau-Veilleux et al. 2020, 2021). One may hypothesize that these investigations may facilitate drug titration with levodopa containing formulations and finally shorten in-patient stays. These measurements to be established in the future may also complement and improve calculations on levodopa equivalents as a measure of disease progression in PD (Jost et al. 2023). This would enable the definition of new, more precise and also more clinical relevant endpoints over trials lasting many years on disease modification. It is well known that enzyme blockers, such as inhibitors of COMT elevate peripheral plasma levodopa, or MAO-B-inhibitors increase central biogenic amines. On the one hand measurement of their plasma concentrations may have limited value for clinicians due to their limited dosing scenarios based on pivotal study results. On the other hand future outcomes from therapeutic drug monitoring in association with an individually determined enzyme blocking potency may open the possibility for an individual optimised dosing, i.e. of rasagiline or safinamide, since safety and tolerability data on higher dosages with 2 mg resp. 200 mg exist, as for example (Stocchi et al. 2012). The same considerations may also be relevant for COMT-inhibition or the use of amantadine in PD patients.

## Conclusion

Therapeutic drug monitoring in PD has not so far not been established. Clinicians determine the dosing and the efficacy of an applied PD drug combination in relation to the clinical response, predominantly the motor behaviour. For instance, among others, an important influencing factor is body weight, or the body mass index. In addition, nutritional aspects, gastrointestinal emptying, consideration of accompanying drug treatment strategies in older patients with multiple health problems other than PD will be a matter of TDM future developments, i.e. to exclude absorption problems, to determine metabolism rate and as a tool to detect compliance problems. Further advantages of TDM in PD were already highlighted in the introduction to this text. Based on actual knowledge and known problems associated with the highly variable plasma levels of antiparkinsonian drugs, TDM in PD patients has the potential to become an important tool for individualized assessment of levodopa plasma bioavailability in particular for the clinical practice. Here TDM will help to determine the aforementioned, still underestimated diversity between original "branded" and the various generic PD drug formulations (see Table 1) in the future. This will perhaps convince authorities, health politicians and financial services that various PD drug preparations, containing the same amount of active ingredients, should not indiscriminately be exchanged for pricing reasons.

## Data Availability

The data included in this review is taken from publishing manuscripts which are cited.
